# Indole-3-Carbinol, a Phytochemical Aryl Hydrocarbon Receptor-Ligand, Induces the mRNA Overexpression of UBE2L3 and Cell Proliferation Arrest

**DOI:** 10.3390/cimb44050139

**Published:** 2022-05-08

**Authors:** Claudia Vanessa Arellano-Gutiérrez, Laura Itzel Quintas-Granados, Hernán Cortés, Manuel González del Carmen, Gerardo Leyva-Gómez, Lilia Patricia Bustamante-Montes, Miguel Rodríguez-Morales, Israel López-Reyes, Juan Ramón Padilla-Mendoza, Lorena Rodríguez-Páez, Gabriela Figueroa-González, Octavio Daniel Reyes-Hernández

**Affiliations:** 1 Laboratorio de Bioquímica Farmacológica, Departamento de Bioquímica, Escuela Nacional de Ciencias Biológicas, Instituto Politécnico Nacional, Ciudad de México 11340, Mexico; c_varella@ymail.com (C.V.A.-G.); lorena_rpaez@yahoo.com.mx (L.R.-P.); 2 Unidad de Estudios Superiores Tultitlán, Universidad Mexiquense del Bicentenario, Estado de Mexico 54910, Mexico; litquingra@gmail.com; 3 Laboratorio de Medicina Genómica, Departamento de Genómica, Instituto Nacional de Rehabilitación Luis Guillermo Ibarra Ibarra, Ciudad de México 14389, Mexico; hcortes_c@hotmail.com; 4 Facultad de Medicina, Universidad Veracruzana, Ciudad Mendoza 94740, Mexico; manueldelcarmen@hotmail.com; 5 Departamento de Farmacia, Facultad de Química, Universidad Nacional Autónoma de México, Ciudad de México 04510, Mexico; gerardoleyva@hotmail.com; 6 Decanatura Ciencias de la Salud, Universidad Autónoma de Guadalajara, Guadalajara 44670, Mexico; patricia.bustamante@edu.uag.mx; 7 Licenciatura en Médico Cirujano-INIES CUI, Ixtlahuaca Jiquipilco, San Pedro, Ixtlahuaca de Rayón 50740, Mexico; leugim_018@hotmail.com; 8 Colegio de Ciencias y Humanidades, Plantel Cuautepec, Universidad Autónoma de la Ciudad de México, Ciudad de México 6720, Mexico; israel.lopez.reyes@uacm.edu.mx; 9 Departamento de Infectómica y Patogénesis Molecular, Centro de Investigación y de Estudios Avanzados (CINVESTAV-IPN), Ciudad de México 07360, Mexico; ibtramon.padilla@outlook.com; 10 Laboratorio de Farmacogenética, UMIEZ, Facultad de Estudios Superiores Zaragoza, Universidad Nacional Autónoma de México, Ciudad de México 09230, Mexico; 11 Laboratorio de Biología Molecular del Cáncer, UMIEZ, Facultad de Estudios Superiores Zaragoza, Universidad Nacional Autónoma de México, Ciudad de México 09230, Mexico

**Keywords:** cervical cancer, indole-3-carbinol, *UBE2L3*, cellular proliferation

## Abstract

Cervical cancer (CC) is one of the most common cancers in women, and is linked to human papillomavirus (HPV) infection. The virus oncoprotein E6 binds to p53, resulting in its degradation and allowing uncontrolled cell proliferation. Meanwhile, the HPV E7 protein maintains host cell differentiation by targeting retinoblastoma tumor suppressor. The host cell can ubiquitinate E6 and E7 through UBE2L3, whose expression depends on the interaction between the aryl hydrocarbon receptor (AhR) with Xenobiotic Responsive Elements (XREs) located in the *UBE2L3* gene promoter. In this study, we used cell culture to determine the effect of indole-3-carbinol (I3C) over cellular viability, apoptosis, cell proliferation, and mRNA levels of *UBE2L3* and *CYP1A1.* In addition, patients’ samples were used to determine the mRNA levels of *UBE2L3* and *CYP1A1* genes. We found that I3C promotes the activation of AhR and decreases cell proliferation, possibly through *UBE2L3* mRNA induction, which would result in the ubiquitination of HPV E7. Since there is a strong requirement for selective and cost-effective cancer treatments, natural AhR ligands such as I3C could represent a novel strategy for cancer treatment.

## 1. Introduction

Cervical cancer (CC) is the most common cancer among women from low-income countries [1]. In 2018, approximately 570,000 cases and 311,000 deaths ranked CC as the fourth most common cancer in women worldwide [2], suggesting that CC remains a significant health issue. Most CC cases (99.7%) are linked to an infection with high-risk human papillomavirus (HPV), which is a widespread sexually transmitted virus [3].

The HPV genome encompasses approximately 8000 base pairs that encode for two structural proteins (L1 and L2), six early proteins, three regulatory proteins (E1, E2, and E4), and three oncoproteins (E5, E6, and E7). Moreover, this genome contains a noncoding regulatory locus control region (LCR) [4]. Primary HPV infections are transitory, but in some cases, these infections may persist and increase the risk of developing pre-cancerous lesions, cervical intraepithelial neoplasia (CIN), and CC [5,6,7]. 

In carcinogenic HPV (types: 16, 18, 31, 33, 35, 39, 45, 51, 52, 56, 58, 59, 68, 73, and 82) [8], the E6 and E7 genes are integrated into the host genome resulting in the overexpression of their mRNAs that may be related with the protein quantity [9,10]. HPV E6 and E7 are oncoproteins involved in the six cancer hallmarks [11]; these possess the ability to bind to cellular proteins and influence the host cell activity [12]. The complex between E6 and E6-associated binding protein (E6AP) binds to UBE3A E2 ubiquitin-conjugating enzyme. This complex produces a structural change in HPV E6 that allows its binding to p53, a tumor suppressor protein, leading to ubiquitination of p53. Thereby, p53 is targeted to the proteasome, where it is degraded, leading to uncontrolled cell proliferation and resistance to cell death [12]. On the other hand, HPV E7 contains a zinc finger domain at the C-terminal, which allows the zinc-dependent dimerization that leads to its interaction with cell cycle regulatory proteins such as p21 and retinoblastoma tumor suppressor (pRb), promoting cellular proliferation (one of the cancer hallmarks) [13]. Thus, the primary function of HPV E7 is to maintain host cell differentiation by targeting pRb, which controls the G1 to S-phase transition [14]. 

The degradation of HPV E7 through the Ubiquitin-Proteasome System (UPS) involves a ubiquitination cascade that transfers ubiquitin to target proteins. E7 is ubiquitinated by the UBE2L3/Cullin 1 (CUL1) complex, and then E7 is degraded via the 26S proteasome; this mechanism has only been described for HPV-16 E7 [14,15]. UBE2L3 (also known as UBCH7, UbcH7, and E2 Ubiquitin-Conjugating Enzyme L3) is a ubiquitin-conjugating protein belonging to the E2 family. 

This protein promotes ubiquitination and degradation of p53 through its interaction with HPV E6/E6AP and UBE2L3, resulting in decreased apoptosis in HeLa cells [16,17]. Thus, HPV E7 and p53 are target proteins of UBE2L3. Interestingly, the expression of *UBE2L3* depends on the interaction between the aryl hydrocarbon receptor (AhR) and Xenobiotic Responsive Elements (XREs), formerly known as Dioxin Response Elements (DRE), located in the *UBE2L3* gene promoter [16]. 

The AhR is a cell cycle mediator that has been associated with several functions in cell proliferation and differentiation, gene regulation, tumor development, and metastasis [18]. Moreover, this receptor is a cytoplasmic transcriptional factor that translocates into the nucleus once it binds to xenobiotic elements such as aromatic ligands. In the nucleus, AhR dimerizes with the AhR nuclear translocator protein (ARNT); this complex binds to XREs from the *UBE2L3* gene promoter resulting in increased levels of *UBE2L3* [16]. Exposure to xenobiotic compounds evolved into an adaptative response involving gene expression induction through AhR, such as the *CYP1A1* gene, which enhances xenobiotic metabolism [19]. Interestingly, environmental toxins such as 2,3,7,8-tetrachlorodibenzo-*p*-dioxin (TCDD) activates a unique AhR-activated/proteasome-dependent pathway which induces degradation of Estrogen Receptor alpha (Erα) and AhR [20]. Contrariwise, the toxic effects of TCDD and other dioxin-like compounds are diminished by AhR. 

Furthermore, some phytochemicals and pharmaceutical compounds are endogenous AhR ligands, including indole-3-carbinol (I3C). I3C and its condensation products have been reported as potential antitumor or carcinogenic compounds [21,22,23,24,25,26]. Their mechanism is not entirely known, but the AhR signaling pathway might represent a potential mediator [27,28]. Moreover, AhR is responsive to agonist ligands such as β-naphthoflavone (BNF), a putative chemotherapeutic synthetic agent with anticancer activity against mammary carcinoma cells [18] and cervical cancer cells [29]. AhR has a high capability of binding several toxins, xenobiotics, and phytochemical compounds, leading to a bifunctional AhR effect: its stimulation causes malignant transformation in specific tissues, while in others, AhR bears a protective role [30,31]. Therefore, identifying non-carcinogenic AhR agonist compounds is crucial for developing alternative cancer treatments. In this respect, I3C is a natural AhR ligand found in cruciferous vegetables such as broccoli, kale, and brussels sprouts, and its dietary inclusion could prevent some diseases. Remarkably, I3C has a suppressive effect on the growth of prostate cancer cell lines [32]. 

Herein, we present functional evidence to increase the understanding of the AhR signaling pathway through I3C. We discovered that I3C induced the expression of *UBE2L3*, which correlated with a decrease in cell proliferation. Therefore, we believe that promoting the expression of *UBE2L3* through AhR natural ligands such as I3C might decrease cell proliferation in CC patients. 

## 2. Materials and Methods

### 2.1. Tissue Samples

A total of 9 cervical samples, diagnosed with CC (*n* = 3), CIN (*n* = 3), and healthy tissues (*n* = 3), were obtained from patients recruited in the Hospital Juarez de Mexico (HJM). This study was performed with the approval of the Research Ethics Committee from HJM (registration number HJM2231/13-B), and all the participants gave their informed consent. 

### 2.2. mRNA Extraction from Patients’ Samples

Cervical tissues were homogenized in 1 mL TRIzol Reagent (Invitrogen, Camarillo, CA, USA) using an electric homogenizer, and RNA isolation was performed according to the manufacturer’s instructions. Then, isolated RNA was dissolved in RNase-free water and stored at −70 °C until use. 

### 2.3. Cell Culture

HeLa cells (ATCC) were cultivated in Dulbecco’s Modified Eagle’s medium-high glucose (Invitrogen, Carlsbad, CA, USA) supplemented with 10% fetal bovine serum (HyClone, Logan, UT, USA) and 1% antibiotic/antimycotic (Invitrogen, Carlsbad, CA, USA) at 37 °C in a 5% CO_2_ atmosphere.

### 2.4. Cell Viability

Several I3C (Sigma-Aldrich, St. Louis, MO, USA; purity ≥ 96%) concentrations (10, 25, 50, 100, 250, 500, and 1000 µM) were used to determine cell viability using the MTT assay, according to a previous report [33]. Results were expressed as the percentage of cell growth to the respective control (untreated cells) for each I3C concentration. Three biological replicates measurements with technical duplicates were performed.

### 2.5. Analysis of Target mRNA

Target mRNAs were quantified by real-time PCR analysis in HeLa cells and densitometric analysis for patient samples (*n* = 3). Total RNA was isolated from HeLa cells treated with I3C (130 and 252 µM), BNF (1 µM; Sigma-Aldrich, St. Louis, MO, USA) (we selected BNF concentration considering its EC50 reported value (10 µM) [18] and the absence of cytotoxic effects), or dimethyl sulfoxide (DMSO; 150 mM; Sigma-Aldrich, St. Louis, MO, USA) using TRIzol reagent. RNA samples (from cellular culture) were analyzed spectrophotometrically and by agarose gel electrophoresis to determine their purity and integrity. Then, 2 µg of total RNA was used to synthesize cDNA using the SuperScript First-Strand Synthesis (Invitrogen, Carlsbad, CA, USA) and oligo dT. Finally, PCR reactions contained 2 µL of cDNA, 1X TaqMan Universal PCR Master Mix (Applied Biosystems), 0.9 µM of primers, and 0.25 µM of probes. Reactions were performed using an ABI PRISM 7000 Sequence Detector System (Applied Biosystems, Branchburg, NJ). The mRNA samples for *UBE2L3*, *CYP1A1*, and 18S ribosomal RNA (rRNA endogenous) were analyzed using the comparative threshold cycle (Ct) method. Probes used for *UBE2L3*, *CYP1A1*, and 18S ribosomal RNA were obtained from Applied Biosystems (Branchburg, NJ, USA) with the identification numbers Hs02382618_s1, Hs00748530_s1, and Hs03928990_g1, respectively. Three biological replicates measurements with technical duplicates were performed. 

Moreover, mRNA from patient samples was used to synthesize cDNA as described above. Then, target genes were amplified by PCR and densitometrically analyzed. Briefly, 15 µL aliquot of each reaction was mixed with running dye solution, loaded into an agarose gel, and electrophoresed. A molecular weight marker specially designed for easy quantification (Gene Ruler, Thermo Scientific™, Waltham, MA, USA) was loaded with the samples. For quantification, the intensity of a specific volume of each band in the samples was compared with the intensity present in the same volume of the molecular weight marker and normalized with the control loading gene. Triplicate analysis for each human sample was performed. 

### 2.6. Flow Cytometry

Cells (3 × 10^5^) treated with camptothecin (CPT, 0.15 µM), BNF (1 µM), or I3C (100 µM) were centrifuged at 1000× *g* for 5 min at room temperature and then washed twice with precooled PBS. We select the CPT concentration considering the reported values used to induce apoptosis effects. The cell pellets were resuspended in 500 µL of binding buffer, followed by the addition of 1.5 µL of Annexin V-fluorescein isothiocyanate (FITC) and 10 μL of propidium iodide (PI; Sigma-Aldrich, Darmstadt, Germany) at a concentration of 50 μg/mL in PBS. Then, samples were incubated in the dark at room temperature for 20 min and analyzed using a flow cytometer (FACS Calibur; BD Biosciences, San Diego, CA, USA) with an excitation wavelength of 488 nm within 1 h. A band-pass filter with a wavelength of 515 nm was applied to detect FITC fluorescence, while another filter with a wavelength of 560 nm was used to detect PI. For each sample, the histograms were normalized to untreated cells, and the apoptotic percentage was estimated considering the number of biological events for each one. Three biological replicates measurements with technical duplicates were performed. 

### 2.7. Colony Formation Assay

Soft agar colony formation assay was used to evaluate cellular proliferation in vitro in the presence of I3C. HeLa cells (9079 cells/well) were inoculated in a 24-well plate with medium DMEM supplemented with 5% fetal bovine serum and containing BNF (1 µM), I3C (100 µM), or vehicle (DMSO, 0.15 µM) and cultured for seven days. Finally, colonies were stained with crystal violet (0.5%), visualized, and counted. Three biological replicates measurements with technical duplicates were performed. 

### 2.8. Statistical Analysis

All data were analyzed using the SPSS software package (SPSS, Chicago, IL, USA). One-way ANOVA combined with the Mann–Whitney test was used to compare differences between different experimental groups, and differences were defined as statistically significant for *p* values < 0.05.

## 3. Results

### 3.1. Effect of I3C on Cells Viability

**Figure 1:**
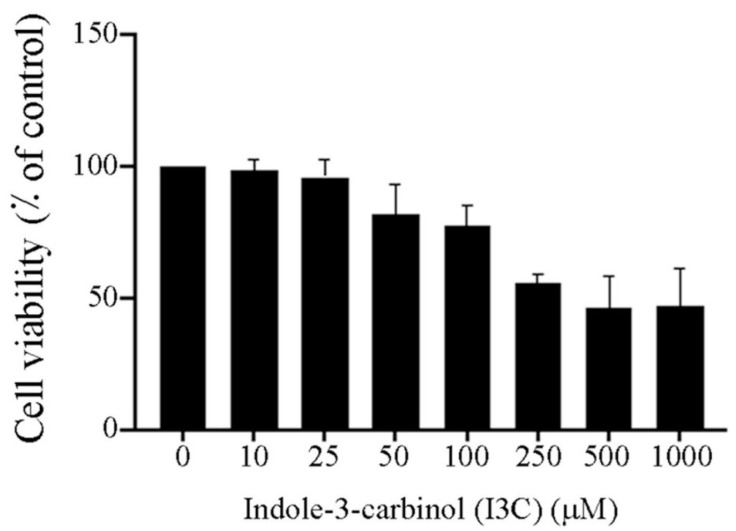
Effect of I3C on the viability of HeLa cells. Cells were incubated with several I3C concentrations (10–1000 µM) for 24 h, and the cell viability was evaluated by MTT colorimetric assay. Data represent the mean ± standard error (SE) and are expressed as a percentage of the respective controls. One-way ANOVA followed by Mann–Whitney test.

First, we studied the effect of I3C on HeLa cells viability (Figure 1). According to our results, the viability of the cells was dose-dependent. However, there were no statistically significant effects at I3C concentrations from 10 µM to 100 µM. On the other hand, above 500 µM, viability of HeLa cells was 50%. Moreover, we calculated a half-maximal effective concentration (EC50) for I3C of about 500 µM in HeLa cells (R^2^ = 0.9342.). All experiments were performed below the EC50 of I3C to ensure that the effects were not due to cytotoxicity. However, all concentrations used activated the AhR, which was corroborated by the expression of *CYP1A1* as a control of gene expression through the AhR.

### 3.2. Effect of I3C on mRNA Levels of UBE2L3

**Figure 2:**
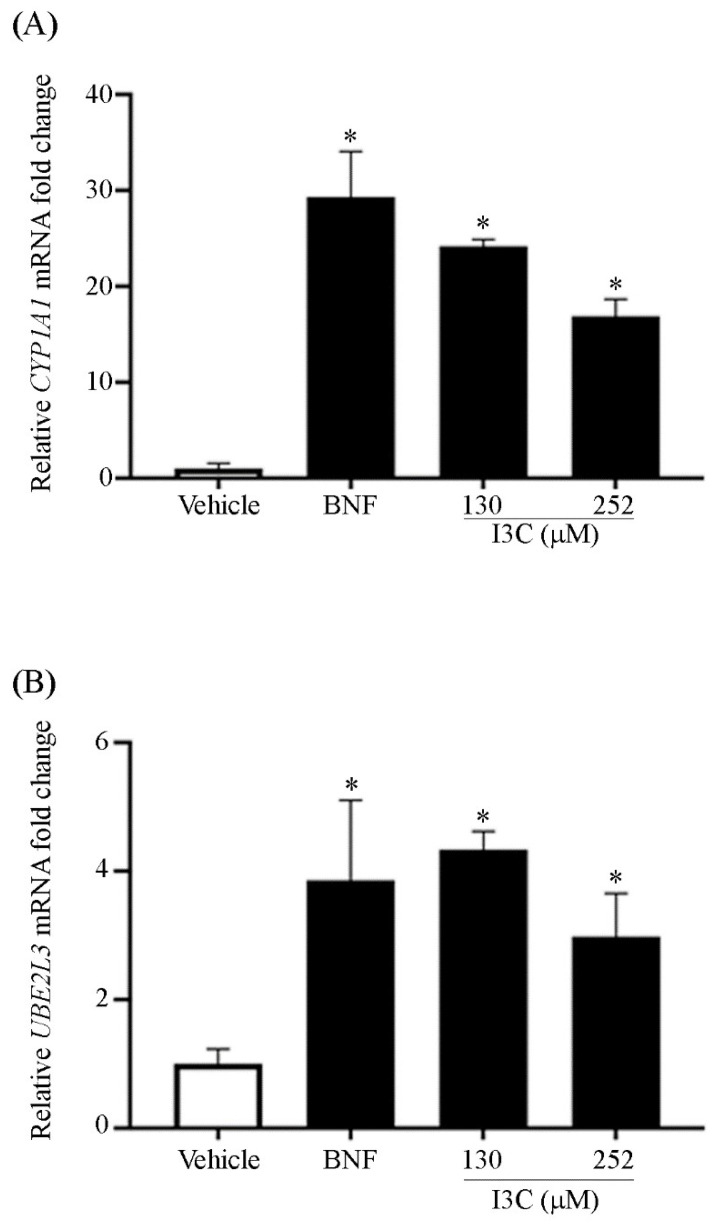
Effect of AhR induction through I3C on the mRNA levels of *UBE2L3* and *CYP1A1*. HeLa cells treated with vehicle (DMSO, 150 mM), BNF (1 µM), and I3C (130 and 252 µM) were used to measure mRNA levels by quantitative RT-PCR of Panel (**A**) *CYP1A1* and Panel (**B**) *UBE2L3* mRNA. Statistically significant differences were found between BNF- or I3C-treated cells compared with the vehicle for *CYP1A1* and *UBE2L3* mRNA (* *p* < 0.05). Bars represent the relative mRNA amount of three independent experiments presented as means ± SE. One-way ANOVA followed by Mann–Whitney test (* *p* < 0.05).

The mRNA levels of *CYP1A1* (Figure 2, panel A) and *UBE2L3* (Figure 2, panel B) were analyzed in HeLa cells treated with BNF (1 µM)) and I3C (130 and 252 µM) using qPCR. According to our results, mRNA levels of *CYP1A1* (as a control of gene expression through AhR) and *UBE2L3* showed a statistically significant increase in cells treated with BNF and I3C. In cells treated with BNF, the amount of *UBE2L3* was 4-fold greater than untreated cells (vehicle), whereas in cells treated with 130 and 252 µM of I3C, the expression on *UBE2L3* was 4.1-fold and 3-fold greater, respectively, than that of control (vehicle). Although apparently target mRNA levels in 252 µM are lower than in 130 µM of I3C, we did not find a statistical difference between both concentrations. In addition, the use of I3C concentrations higher than 252 µM, may result in fewer target mRNA levels due to the low water solubility of I3C.

These findings suggest that the binding of BNF or I3C to AhR up-regulates the mRNA transcription not only of *CYP1A1* but *UBE2L3*. In our study model, increased *CYP1A1* levels corroborated that AhR was activated by its synthetic (BNF) and natural (I3C) agonist ligands (Figure 2, Panel A). In I3C-treated cells, *UBE2L3* levels were statistically higher compared with the control group (*p* < 0.05). Similar behavior was observed in BNF treatment (Figure 2, Panel B), suggesting that the activation of AhR resulted in *UBE2L3* induction. 

### 3.3. Effect of I3C on Apoptosis and Proliferation of HeLa Cells

**Figure 3:**
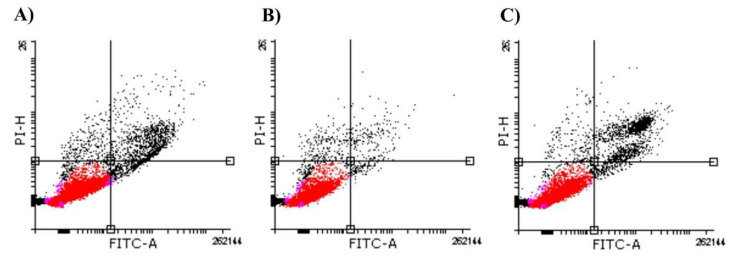
Effect of I3C on the apoptosis rate. Representative histograms (normalized to untreated cells) of HeLa cells cultures treated with camptothecin (CPT, 0.15 µM) (control) (Panel (**A**)), BNF (1 µM) (Panel (**B**)), or I3C (100 µM) (Panel (**C**)). Viable cells are in the lower left-hand quadrant (red). The right lower quadrant demonstrates apoptotic cells. Necrotic cells are in the upper left-hand quadrant. FITC, fluorescein isothiocyanate; PI, propidium iodide.

Since the ubiquitination of HPV E7 and p53 via UBE2L3 might affect some cancer hallmarks, we investigated HeLa cells’ apoptosis and proliferation rates. Apoptosis data are shown in a double-variable flow scatter diagram divided into quadrants (Figure 3). Regardless of the variations in the number of events considered for each of the treatments, the percentages of apoptotic cells (right lower quadrant) were less than 1% for HeLa cells treated with BNF (Figure 3, Panel B) or I3C (Figure 3, Panel C). Statistical analysis of the apoptosis ratio (data not shown) indicates no difference between BNF and I3C samples (*p* = 0.184), suggesting that I3C had no direct effect exclusively on apoptosis. 

**Figure 4:**
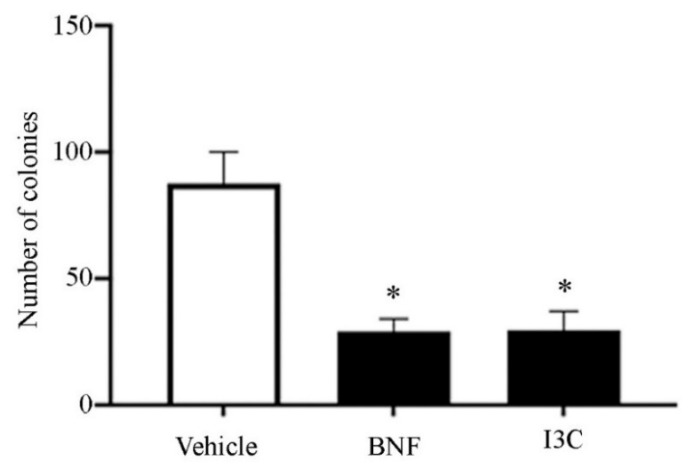
Effect of *UBE2L3* overexpression on cell proliferation in vitro. HeLa cells were cultured in a soft agar medium in the presence of I3C (100 µM), BNF (1 µM), or vehicle (DMSO, 0.15 µM) for colony formation assay. Bars represent the mean colony count of three independent experiments in the presence of I3C, BNF, or vehicle ± SE. One-way ANOVA followed by Mann–Whitney test (* *p* < 0.05).

Furthermore, we assessed the proliferation rate through the number of colonies counted in treated HeLa cells (Figure 4). We observed a statistically significant difference between I3C and BNF treatments compared with vehicle (*p* > 0.05), but no statistical differences between the colonies counted in the presence of I3C compared with BNF. These data suggested that I3C treatment had an important role in inhibiting the proliferation of cancer cells.

### 3.4. Quantification of UBE2L3 mRNA in Patient Samples

**Figure 5:**
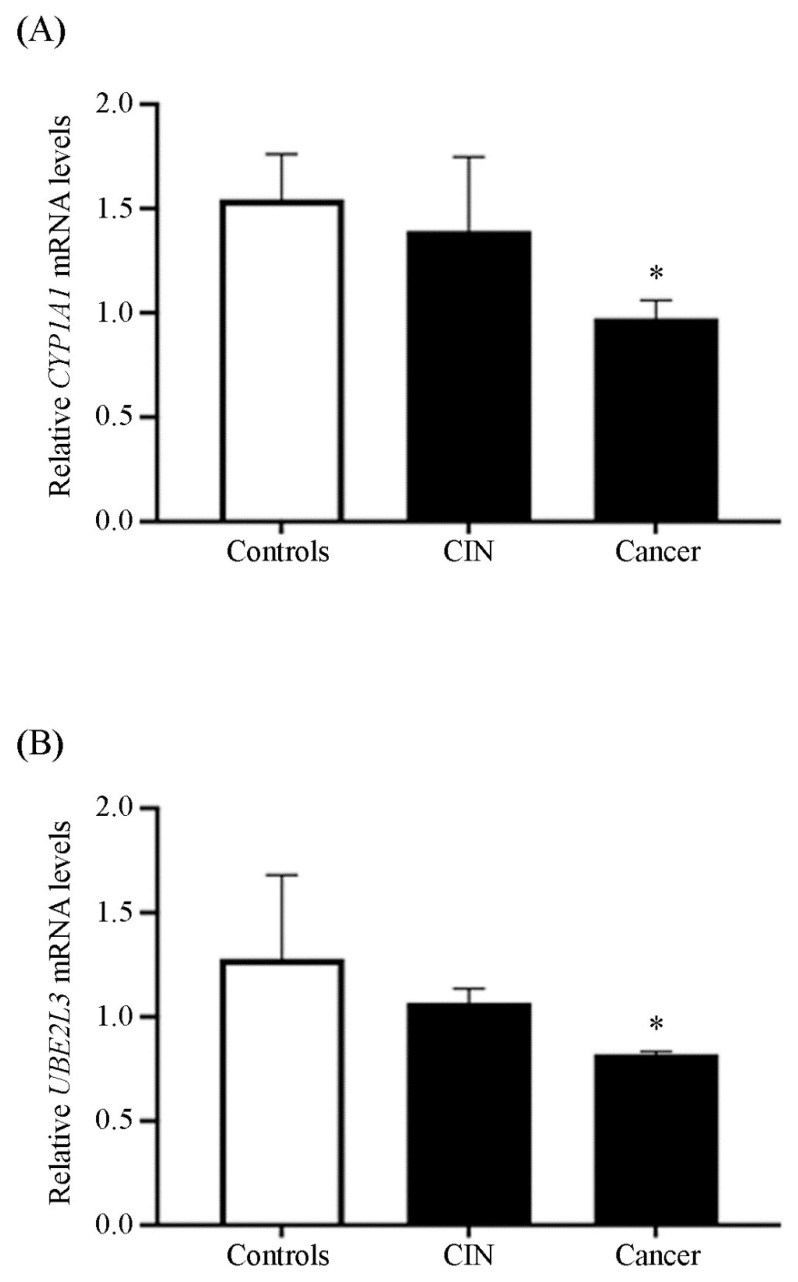
*UBE2L3* mRNA relative levels in human tissue samples. Samples of patients with cervical cancer or cervical intraepithelial neoplasia (CIN) and healthy cervical samples (controls) were used to determine the Panel (**A**) *CYP1A1* and Panel (**B**) *UBE2L3* mRNA levels. Bars represent the mean of 3 patients for each group ± SE. One-way ANOVA followed by Mann–Whitney test (* *p* < 0.05).

Additionally, we wished to examine whether our proposed model was feasible in a live environment. Patients diagnosed with CC, CIN, and healthy participants (controls) not under treatment or who do not consume I3C were considered for this research. Agarose gel electrophoretic profile of RT-PCR samples used for semi-quantitative analysis of *UBE2L3* is shown in Supplemental Figure S1. We quantified *CYP1A1* and *UBE2L3* mRNAs in samples from patients (Figure 5) using endpoint PCR and analyzed the data. We observed that *CYP1A1* mRNA in CC samples exhibited a decrease of 33% in comparison with controls (*p* > 0.05), while in CIN samples, *CYP1A1* mRNA level did not show a statistical difference compared with controls (Figure 5, Panel A). On the other hand, the *UBE2L3* mRNA amount in CC samples decreased to half of the level found in the control group (*p* > 0.05) (Figure 5, Panel B). In contrast, *UBE2L3* mRNA level exhibited no difference in controls and CIN samples. Furthermore, there was no statistical difference between the amount of UBE2L3 protein in samples from CC patients compared with controls (data not shown). 

## 4. Discussion

In this article, we showed some evidence that I3C may promote the activation of AhR, inducing the expression of *UBE2L3* and decreasing cell proliferation. Since there is an imperative need for selective and inexpensive medications against CC, natural AhR ligands such as I3C might represent a novel and exciting alternative. In the stomach, the low-pH environment causes an acid condensation of I3C, generating condensation products such as indolo [3,2-*b*] carbazole (ICZ) that are related to the biological effects of dietary I3C [34]. In vivo, condensation products of I3C appear to be potent agonists of AhR [35].

Our results revealed that I3C concentrations ranging from 10 to 250 µM did not affect HeLa cells’ cellular viability; however, I3C concentrations above 250 µM diminished the cell viability by 50%. Interestingly, low concentrations of I3C decreased the viability of prostate cancer cells that express androgen receptors [36,37], suggesting that cellular responses to I3C depend on biological and chemical factors such as cell type, profiles of genomic expression, time, and dosage. In this way, I3C exhibits anticancer effects on several colorectal cancer cell lines, causing cell death through AhR activation. Likewise, I3C and its derivatives suppress the proliferation of breast [25,38,39,40,41,42], colon [43,44,45], prostate [46,47,48], and endometrium [49] cancer lines. Furthermore, I3C induces apoptosis in osteosarcoma [50], breast [51], and melanoma cells [52]. I3C efficacy depends on its dynamic molecular interaction and the activation of multiple signaling targets [28]. Intrinsic I3C instability generates oligomeric condensation products with pharmacological activities [28], such as apoptosis and cell cycle arrest in prostate and pancreatic cancer cells [36,53]. Furthermore, in neutral culture media, I3C underwent dimerization into 3,3′-Diindolylmethane (DIM) in 24 h at 37 °C [54]. We considered that some amount of DIM may be present in our experiments, but its activity might enhance the cancer prevention effects of I3C.

In this work, we used *CYP1A1* as an internal control to induce the classical activation cascade of AhR and compared it with our results. Remarkably, despite their binding capabilities xenobiotics activate *CYP1A1* gene expression [55]. Moreover, metabolic products of phytochemicals could have weaker or higher affinities to AhR, but even a weak inducer compound may potentially signal. Diverse compounds might stimulate AhR and activate *CYP1A1*, 1A2, and 1B1 [56]; thus, different AhR ligands might be exploited as targets for the development of novel treatments. It is well known that BNF, a synthetic agonist of AhR, increases the mRNA levels of *CYP1A1*, and according to our results, BNF also increases *UBE2L3* levels. However, BNF has been associated with mutagenic and carcinogenic activities [57]. Since I3C is a weak ligand of AhR, we used high amounts of I3C to exacerbate its effects for target gene induction; however, we are not suggesting that these doses might be used for clinical assays. Therefore, we explored the basics of I3C as an anticancer preventive strategy. A previous report indicates that I3C activates AhR and promotes the expression of AhR target genes [26].

Our findings showed that I3C promoted the expression of *CYP1A1* and *UBE2L3* mRNA. Moreover, I3C increased UBE2L3 mRNA levels in the same manner as BNF. The interaction of AhR ligands towards the AhR ligand-binding domain was reported using molecular modeling, molecular docking, and molecular dynamic simulations. According to these results, AhR ligands showed different flexibility to adopt a favorable conformation during its interaction [58]. Our findings suggest that this natural phytochemical compound is capable of stimulating the expression of *UBE2L3*. Although synthetic ligands promote a higher level of expression of *UBE2L3*, most of them present adverse side effects that are not reported for I3C. Although we demonstrate sufficiently strong evidence, the major weakness of our research is that we do not yet know whether the effects of I3C on *UBE2L3* are *AhR*-dependent. However, experiments are underway to find an answer.

On the other hand, viral oncoproteins HPV E6 and E7 induce cancer hallmarks, generally uncontrolled cellular proliferation and evasion of apoptosis [11], targeting growth suppressors such as p53 and pRb. In this manner, HPV E6 has a crucial effect on continuous cellular proliferation through apoptosis evasion via p53 [12,59]. In contrast, the primary function of HPV E7 in CC is regulating host cell differentiation, which is reached by targeting pRb that controls the G1 to S-phase transition [14]. In I3C-treated HeLa cells, the complex AhR-I3C is assembled in the cytoplasm. It is then translocated into the nucleus, where it binds to ARNT, allowing its binding to XREs located in the *UBE2L3* gene promoter, resulting in increased levels of UBE2L3 [16]. Nuclear AhR localization results in a higher tumor grade, more poorly differentiated cells, and poor prognosis, suggesting that AhR may contribute to increasing cancer aggression [60,61,62,63,64].

**Figure 6:**
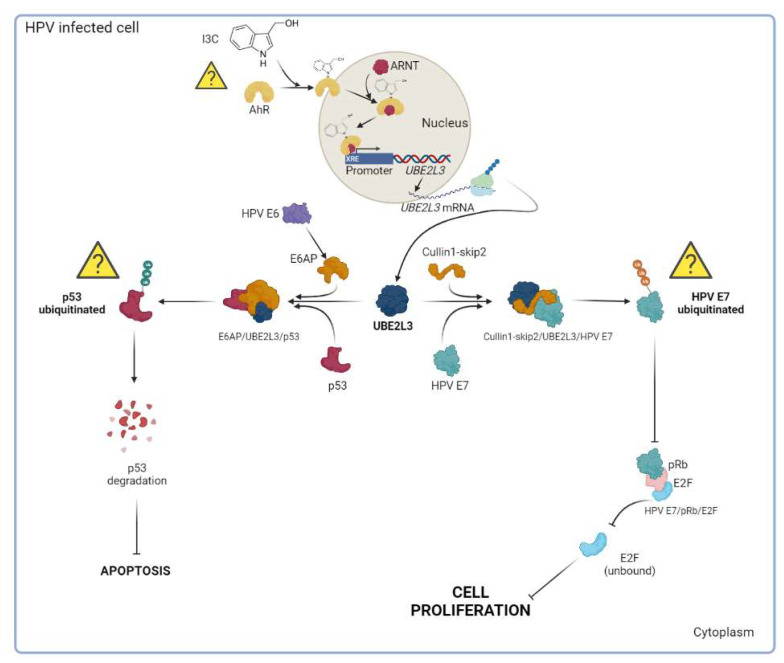
Hypothetical model of I3C mechanism in HeLa cells. AhR ligands such as I3C bind to the AhR protein in the cytoplasm. Then, the AhR-I3C complex is translocated to the nucleus and binds to Xenobiotic Responsive Elements (XREs) in the *UBE2L3* gene promoter, leading to an increase in the *UBE2L3* expression (at transcriptional and translational levels). Subsequently, UBE2L3 might ubiquitinate HPV E7 or p53 in HPV-infected cells. In the presence of high levels of *UBE2L3*, as a result of AhR activation by I3C, p53 is not ubiquitinated because the trimeric complex HPV E6/E6AP/p53 is not assembled. Therefore, p53 is not degraded, and apoptotic levels remain unchanged. On the other hand, cell proliferation decreased, suggesting that I3C-induced overexpression of *UBE2L3* resulted in HPV E7 ubiquitination leading to the arrest of cell proliferation. Furthermore, the complex pRb/E2F was not dissociated in the presence of ubiquitinated HPV E7; therefore, cell proliferation mediated by unbound E2F was blocked. Our results suggest that the treatment of HeLa cells with I3C resulted in cell proliferation inhibition rather than a change in the apoptosis rate. Question marks indicate that more conclusive evidence is needed.

The *UBE2L3* gene overexpression induced by I3C might lead to the ubiquitination of p53 or E7 (Figure 6, question marks within triangles), which are substrates of *UBE2L3* [16,17]. Since we observed a significant decrease in cell proliferation rather than a change in cell apoptosis, our findings suggest that I3C may substantially affect the ubiquitination of HPV E7 rather than p53.

In this regard, if the *UBE2L3* overexpression resulted in p53 ubiquitination, then the apoptotic levels were changed due to p53 degradation. However, the apoptosis rate was similar when comparing I3C, CPT, or BNF treatments, suggesting that the overexpression of *UBE2L3* via I3C has no significant effect on the p53 ubiquitination, and therefore, on cellular apoptosis. On the other hand, our findings suggested that the *UBE2L3* overexpression led to the HPV E7 ubiquitination and inhibition of cell proliferation. This result implies that the complex Rb/E2F was not dissociated in the presence of ubiquitinated HPV E7; therefore, unbound E2F-mediated cell proliferation was blocked in I3C-treated cells. The interaction of pRb with E2F is a critical checkpoint in the G1-S phase transition of the cellular cycle. If the pRb/E2F complex remains bound, the genes required in the S phase, such as cyclin E, cyclin A, and p16INK4A (an inhibitor of CDK4/6), are not transcribed. In CC, HPV E7 targets pRb for ubiquitination, releasing E2F from the pRb/E2F complex, forcing the cells into uncontrollable cell proliferation [65].

It has been reported that I3C causes G1 arrest in breast and prostate cancer cells [66,67], involving the up-regulation of CDK inhibitors and the down-regulation of cyclins [68,69]. Furthermore, I3C promotes selective alterations in cyclin E composition, size distribution, and subcellular localization leading to an inhibition of CDK2 kinase activity [70]. Therefore, I3C decreases pRb phosphorylation, leading to pRb binding to the E2F transcriptional factor resulting in G1 arrest [28].

Furthermore, we showed an approach of what might occur in a carcinogenesis environment. In CC and CIN patient samples, the levels of *CYP1A1* decreased compared with controls, suggesting a possible relationship between these levels and the progression of pathology. The mRNA expression patterns of *CYP1A1* in cancer tissues showed high variability, probably due to poor tissue integrity, freezing, or preservation agents, among others. For example, the expression profile of *CYP1A1* in lung tissues is influenced by the induction of enzymes through environmental chemical exposure [71]. The expression of *CYP1A1* is suppressed in normal physiological conditions, but AhR-ligands induce its transcription. However, *CYP1A1* is constitutively expressed during breast cancer, contributing to its progression [72,73,74]. Therefore, *CYP1A1* expression might be associated with cancer pathophysiology in CC tissues.

Finally, higher *UBE2L3* mRNA levels have been associated with tumor size, clinical grade, and prognosis of hepatocellular carcinoma, but its depletion is related to proliferation inhibition and apoptosis induction [75]. Here, we found that *UBE2L3* mRNA levels decreased in CIN and CC samples, suggesting a possible relationship between CC progression and *UBE2L3* gene expression. Therefore, we considered that since the *UBE2L3* mRNA expression is decreased in CIN and CC, I3C consumption might increase its levels. Remarkably, although all patients had not received any treatment or consumed I3C, basal levels of *UBE2L3* were observed, suggesting a possible link between tumorigenesis and *UBE2L3* expression. Interestingly, I3C significantly increased the *UBE2L3* mRNA levels in vitro, possibly leading to cell proliferation inhibition. However, more evidence is needed to demonstrate the role of I3C in vivo. Our results show the potential utility of AhR natural ligands, such as I3C, leading to cell proliferation arrest as possible therapeutic drugs. A hypothetical model of the effect of I3C in cancer cells is shown in Figure 6.

## 5. Conclusions

The genus Brassica contains I3C, a natural compound that induce the expression of *CYP1A1* and *UBE2L3* mRNA made possible through AhR. However, further experiments are need to determinate whether the effects of I3C on *UBE2L3* are AhR-dependent. Moreover, evidence presented here provides insights into the modulation of cell growth and division through I3C, suggesting that I3C may be involved in suppressing cell proliferation of cervical cancer cells in vitro.

## Data Availability

To obtain access to the data that served to support the results found in this study, contact the corresponding author.

## Supplementary Materials

The following supporting information can be downloaded at: https://www.mdpi.com/article/10.3390/cimb44050139/s1.
