# Supplementary figures and images for: Indole-3-Carbinol, a Phytochemical Aryl Hydrocarbon Receptor-Ligand, Induces the mRNA Overexpression of UBE2L3 and Cell Proliferation Arrest
Source: Curr Issues Mol Biol. 2022 May 8;44(5):2054–68. doi: 10.3390/cimb44050139 (PMC9164055; doi:10.3390/cimb44050139)

## Patients' tumor samples

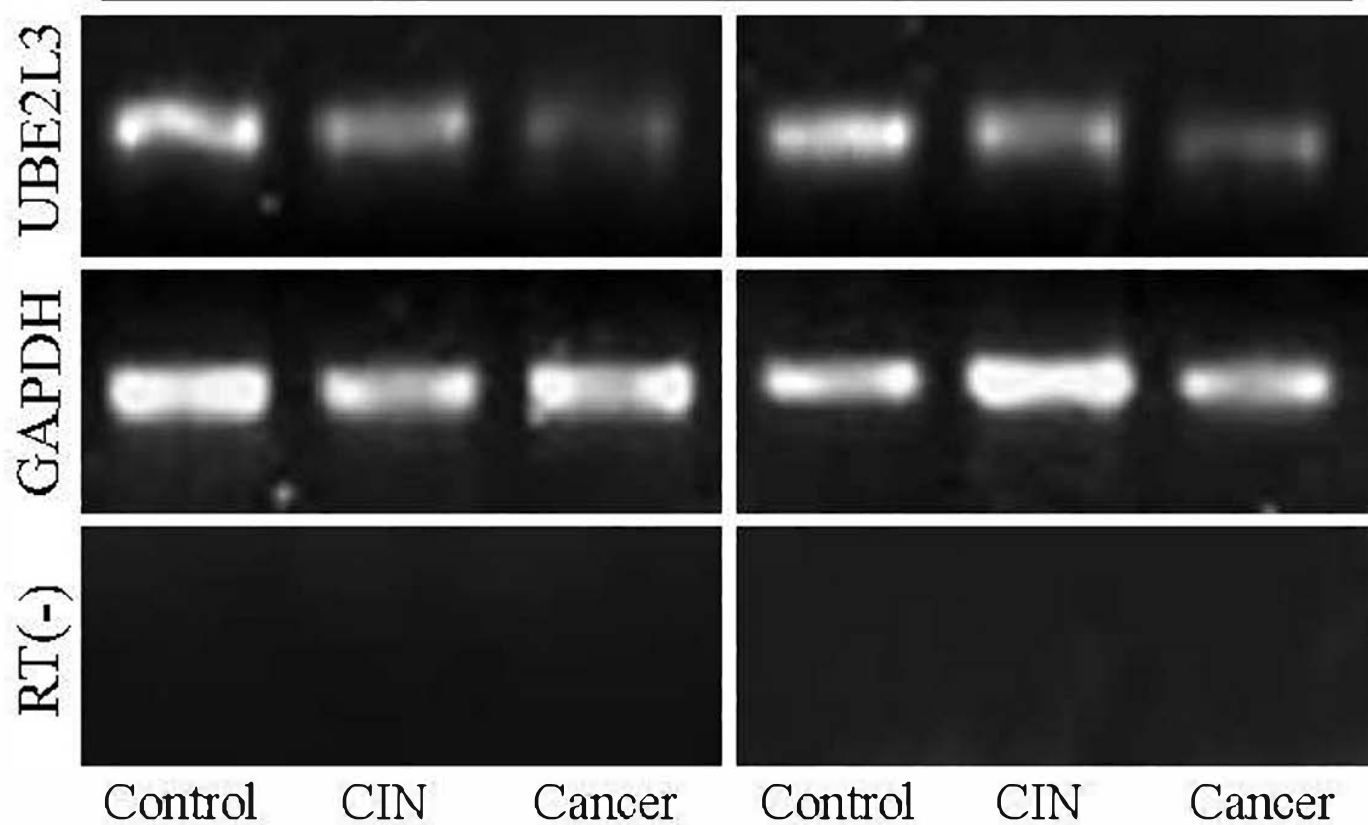

**Figure s1**

Supplement: Supplementary file 1 [file cimb-44-00139-s001.zip › cimb-1631578-supplementary.pdf]
